# Human Prion Disorders: Review of the Current Literature and a Twenty-Year Experience of the National Surveillance Center in the Czech Republic

**DOI:** 10.3390/diagnostics11101821

**Published:** 2021-10-01

**Authors:** Nikol Jankovska, Robert Rusina, Magdalena Bruzova, Eva Parobkova, Tomas Olejar, Radoslav Matej

**Affiliations:** 1 Department of Pathology and Molecular Medicine, Third Faculty of Medicine, Charles University and Thomayer University Hospital, 14059 Prague, Czech Republic; magdalena.bruzova@ftn.cz (M.B.); eva.parobkova@ftn.cz (E.P.); tomas.olejar@ftn.cz (T.O.); radoslav.matej@ftn.cz (R.M.); 2 Department of Neurology, Third Faculty of Medicine, Charles University and Thomayer University Hospital, 14059 Prague, Czech Republic; robert.rusina@ftn.cz; 3 Department of Pathology, First Faculty of Medicine, Charles University, and General University Hospital, 12800 Prague, Czech Republic; 4 Department of Pathology, Third Faculty of Medicine, Charles University, and University Hospital Kralovske Vinohrady, 10034 Prague, Czech Republic

**Keywords:** transmissible spongiform encephalopathies, prion protein, Creutzfeldt–Jakob disease, Gerstmann–Sträussler–Scheinker syndrome, corneal donor

## Abstract

Human prion disorders (transmissible spongiform encephalopathies, TSEs) are unique, progressive, and fatal neurodegenerative diseases caused by aggregation of misfolded prion protein in neuronal tissue. Due to the potential transmission, human TSEs are under active surveillance in a majority of countries; in the Czech Republic data are centralized at the National surveillance center (NRL) which has a clinical and a neuropathological subdivision. The aim of our article is to review current knowledge about human TSEs and summarize the experience of active surveillance of human prion diseases in the Czech Republic during the last 20 years. Possible or probable TSEs undergo a mandatory autopsy using a standardized protocol. From 2001 to 2020, 305 cases of sporadic and genetic TSEs including 8 rare cases of Gerstmann–Sträussler–Scheinker syndrome (GSS) were confirmed. Additionally, in the Czech Republic, brain samples from all corneal donors have been tested by the NRL immunology laboratory to increase the safety of corneal transplants since January 2007. All tested 6590 corneal donor brain tissue samples were negative for prion protein deposits. Moreover, the routine use of diagnostic criteria including biomarkers are robust enough, and not even the COVID-19 pandemic has negatively impacted TSEs surveillance in the Czech Republic.

## 1. Background—Human Prion Diseases in Review

Prion diseases are transmissible, progressive, and fatal neurodegenerative disorders associated with the aggregation of a misfolded prion protein (PrP) [1]. Human transmissible spongiform encephalopathies (TSEs) include Creutzfeldt–Jakob disease (CJD), Gerstmann–Sträussler–Scheinker syndrome (GSS), kuru, and fatal familial insomnia (FFI) [2].

The cellular prion protein (PrP^C^) functions as a glycolipid-anchored cell membrane sialoglycoprotein localized in presynaptic membranes that has neuroprotective [3] and pro-myelinating [4] roles. Additionally, it participates in neurotransmission, zinc and copper transport, and calcium homeostasis [5,6,7]. Moreover, under laboratory conditions, PrP^C^ promotes greater neuronal resistance after an ischemic cerebral insult [8,9].

All human prion diseases are associated with a pathological self-replicating [10] conformation of PrP, the most fundamental of which is the change of the PrP tertiary structure, by post-translational processes, into the predominant β-sheet pattern [11]. The process results in the formation of a markedly hydrophobic form of PrP with a clear tendency toward aggregation [12], subsequent oligomerization, and formation of amyloid fibrils [13]. The pathological PrP^Sc^ (scrapie isoform of the prion protein) aggregates that arise from this process are extremely resistant to physical and chemical changes, and unlike most proteins, these molecules are not denatured by boiling [14].

Human prion diseases [15] are defined as transmissible and rapidly progressive [16] degenerative diseases of the central nervous system caused by an accumulation of pathologically conformed PrP [17].

Historically, the first mention of CJD comes from 1920 [18] and 1921 [19,20,21], when neurologist Hans Gerhard Creutzfeldt and neuropathologist Alfons Maria Jakob described a “nosologically very closely connected if not identical affection” of several patients. Kuru was given attention in the first half of the 20th century as it was described in Papua New Guinea among cannibalistic tribes; the disease is currently considered extinct [22]. The most recent form of TSE, i.e., Variant CJD (vCJD), was first identified in 1996 in the United Kingdom [23,24], which to this day remains the country with the highest number of cases (174) out of a total of 232 worldwide [25]. The last three known cases of vCJD came from Italy and France. An Italian patient with occupational contact with vCJD but without evidence of a laboratory incident died in 2016, and a French laboratory worker died in 2018, 7.5 years after a cutting incident with BSE transgenic mice contaminated instrument [26]. The last case that is under investigation appeared in 2021, when CJD was diagnosed in a retired French laboratory worker which led to 3-month moratorium on the study of prions in France [27].

### 1.1. Sporadic Human Prion Diseases

Creutzfeldt–Jakob disease (CJD) is the most common human prion disease [15]. The neuropathological characterization of CJD is spongiform encephalopathy in cerebral and/or cerebellar cortex and/or subcortical grey matter. It can also be described as encephalopathy with PrP immunoreactivity (plaque and/or diffuse synaptic and/or patchy/perivacuolar type) [28]. Three types, the most common being sporadic (sCJD), followed by genetic (gCJD) and acquired that can be further subdivided into iatrogenic (iCJD) [29], and Variant (vCJD) [30], are distinguished according to their different aetiologies [31]. Using Western blot, we can distinguish between PrP^Sc^ type 1 and 2 [32].

#### 1.1.1. Sporadic Creutzfeldt-Jakob Disease

The sporadic CJD begins with an accidental conversion of physiological PrP^C^ to pathologically conformed PrP^Sc^, which occurs in about 85% of CJD cases [33]. The worldwide incidence of sCJD is reported to be one to two cases per million [34]. Unlike vCJD, clinical signs and neuropathological findings differ from case to case, which is probably caused by different molecular phenotypes [35].

According to the diagnostic criteria recently published by Watson et al. [36] (see Table 1), three conditions can be distinguished: possible CJD (clinical presentation only and exclusion of distinct aetiologies, i.e., tumor, cerebrovascular lesions, autoimmune disorders, neuroinfection, neurodegenerative dementia, etc.), probable CJD (clinical presentation plus biomarkers: protein 14-3-3, magnetic resonance imaging (MRI) [37,38], electroencephalography (EEG) [39] with indicated sensitivity 67% and specificity 86% [40], and RT-QuIC) and definite CJD (neuropathologically confirmed).

The disease usually lasts for a few months, generally less than one year. The duration of the disease must be less than two years; longer durations are an exclusionary clinical criterion for possible sCJD [42].

Due to the risk of iatrogenic transmission, brain biopsy is only applicable in specific cases where a definitive diagnosis is critical. Arguments against brain biopsies in suspected CJD cases include the high probability of a unconclusive result [43]; additionally, it does not affect patient treatment even when the biopsy confirms clinical suspicions [44].

#### 1.1.2. Sporadic Fatal Insomnia

Sporadic fatal insomnia (sFI) is defined as a rapidly progressive neurodegenerative disease with a clinical phenotype very similar to the fatal familial insomnia characterized by neurological and cognitive deterioration along with severe sleep impairment [45], transient diplopia [46] and cerebellar dysfunction [47], followed by dysautonomia, coma and death [45]. These patients are rare codon 129 methionine homozygotes with PrP^Sc^ type 2 and predominant thalamic involvement [48]. Microscopically, slight spongiform degeneration but severe neuronal loss with gliosis is present in thalamus and inferior olives, although immunohistochemical detection of PrP mostly shows focal or no positivity [48]. The distribution of the PrP^Sc^-immunoreactive structures is similar in familial and sporadic form [49,50,51]. The features useful to distinguish sporadic versus familial form are the absence of a family history and the characteristic Asp178Asn *PRNP* mutation [49,50]. 

#### 1.1.3. Variably Protease-Sensitive Prionopathy

Variably protease-sensitive prionopathy (VPSPr) is a relatively recently described prion disease identified in 2008 [52,53]. VPSPr is considered a sporadic form of human prion disease, and patients with all types of polymorphisms at codon 129, however, with a predominance of VV homozygotes, are reported [54]. At the same time, it is stated that VV homozygotes show more developed neuropathological manifestation in the form of plaques than MM homozygotes or MV heterozygotes. The median duration of the disease is 2 years with the clinical predominance of psychiatric signs [53], aphasia, ataxia, and parkinsonian syndrome [54] or cognitive decline [53]. Neuropathologically, VPSPr is characterized by mild spongiform degeneration [53] lacking areas of confluent spongiform transformation [55], PrP-immunoreactive “microplaques”, plus plaque-like deposits [53]. 

### 1.2. Acquired CJD 

#### 1.2.1. Iatrogenic, Accidentally Transmitted CJD

The iatrogenic form arises during medical or surgical procedures during which pathologically conformed prions are transferred [56]. Iatrogenic CJD is extremely rare, accounting for less than 1% of all CJD cases [57]. Transmission after dura mater grafting, from surgical instruments, after corneal transplantation, deep EEG electrode insertion, human growth hormone and gonadotropin treatment have been described [58,59,60] and more than 492 cases have been reported [58]. The incubation time varies widely based on the form of inoculation. Those infected via intracerebral electrodes had an incubation period of 16–28 months, whereas patients infected via peripheral growth hormone injections had a latency of between 5 and 30 years [35]. Patients with iCJD usually present with gait abnormalities and ataxia [58]. Specific diagnostic criteria also exist for iCJD (see Table 2).

#### 1.2.2. Variant CJD

The Variant CJD (vCJD) is associated with the consumption of BSE-agent contaminated products. The clinical presentation initially includes psychiatric and behavioral symptoms, with painful paresthesia or dysesthesia [58]; ataxia and dementia develop later [35]. In contrast to sCJD and gCJD cases, EEG usually lacks the periodic pattern [61], the duration of the disease is usually longer (on average 13–14 months), and florid plaques are often present in the neuropathological findings [62]. The characteristic finding on MRI is an increased bilateral pulvinar signal (with indicated sensitivity 78% and specificity 100%). The thalamic and periaqueductal grey matter high signal, and the remarkable absence of cerebral atrophy are described [63]. In contrast to sporadic and genetic forms of CJD, the presence of PrP^Sc^ demonstrated both immunohistochemically and by Western blot was proven in all types of lymphoid tissue (tonsils, lymph nodes, and spleen) in all vCJD cases [64]. For this reason, tonsil biopsy may be used in suspected vCJD cases with corresponding clinical presentation and MRI lacking bilateral pulvinar high signal to confirm probable vCJD diagnosis [25]. With one exception (one patient with MV genotype) [65], all patients with vCJD were MM homozygotes [66], in addition, PrP^Sc^ type 2 was present in all cases [67]. Interestingly, there are three probable cases of CJD transmission via blood transfusions [59,60] from a donor suffering from vCJD; for this reason, there is a ban on donors who lived in the United Kingdom during the BSE epidemic [68]. Moreover, there are three recently known (2016, 2018, and 2021 which is under investigation) cases of CJD in former laboratory workers with occupational contact with BSE-infected brain tissue [28]. The diagnostic criteria are summarized below (Table 3). 

#### 1.2.3. Kuru

Kuru was defined as a neurodegenerative, non-inflammatory infectious disease [69]. Kuru began to appear around 1900 in Papua New Guinea among cannibalistic tribes and the incidence subsequently escalated between 1940–1950 [69,70]. Cerebellar ataxia, tremor, and extrapyramidal symptoms such as chorea and athetosis [66,69,70,71] were typical symptoms of the majority of patients although neuropathological findings varied [72]. No cognitive impairment was present [58]. The neuropathological reports describe myelin and neuronal degeneration (with a maximum in pontine nuclei, cerebellum and basal ganglia), proliferation of microglia and astroglia [72], mononuclear perivascular infiltration, cuffing [73], spongiform transformation [74], shrunken neurons with dispersion of Nissl substance with intracytoplasmic vacuoles, and vacuolated cerebellar Purkinje cells and striatal neurons [75]. Amyloid “kuru” plaques were recorded in 50-75% [74,75] of the examined brains. Currently, kuru disease is considered extinct [22].

### 1.3. Inherited Prion Diseases

#### 1.3.1. Genetic CJD

The genetic/familial form is conditioned by the presence of an inherited mutation in the *PRNP* gene, which occurs in 10–15% of CJD cases [76]. It is more appropriate to use the term genetic, as not every patient has known positive family history. There are more than 50 known mutations in this gene [35]. In the Czech Republic, the majority of genetic forms involve an E200K mutation, which is also the most common mutation in Europe [77,78] followed by the V210I and D178N mutations. The most common mutations in Japan are V180I, E200K, and M232R, sorted by frequency [77,79]. The D178N mutation is relatively common in certain countries in Western Europe [77,78], i.e., Netherlands, France, United Kingdom, Finland, and Hungary. Dementia is clinically described with other psychiatric changes along with ataxia and myoclonus, however, gaze palsies and neuropathies are rare [58]. The disease penetrance varies from 60 to 100%, relative to population [80]. Similar to sCJD, gCJD has its diagnostic criteria (see Table 4).

The course of the disease is usually longer; the 2-year disease duration limitation for sporadic CJD is not applicable in gCJD.

#### 1.3.2. Gerstmann–Sträussler–Scheinker Syndrome 

Gerstmann–Sträussler–Scheinker syndrome (GSS) is defined as a slowly progressive hereditary autosomal dominant neurodegenerative disease [81] or an encephalo(myelo)pathy with multicentric PrP plaques [28] in the cerebral and cerebellar cortex and basal ganglia [82,83]. It is the multicentric plaques that are the neuropathological hallmark of GSS, although the pattern differs among families [81]. GSS is usually manifested by cerebellar ataxia and slowly progressive dementia [84]; nevertheless, extrapyramidal symptoms, vision and hearing impairment, myoclonus, spastic paraparesis, and hyporeflexia or areflexia in the lower extremities have also been reported as common symptoms [84]. Four distinct clinical subtypes among cases with P102L mutation can be distinguished: typical GSS; GSS with areflexia and paresthesia; pure dementia GSS; and Creutzfeldt–Jakob disease-like GSS [85].

In addition, GSS was the first human TSE with a known *PRNP* mutation [81], which include point mutations at codons 102, 105, 117, 131, 145, 187, 198, 202, 212, 217, and 232 [81] or octapeptide repeat insertions (OPRI) counting 1–9 of 24 base pair multiples [86]. In the Czech Republic, the P102L mutation is the most common.

#### 1.3.3. Fatal Familial Insomnia

Fatal familial insomnia (FFI) is an autosomal dominant inherited disease caused by a mutation D178N in the *PRNP* gene associated with the presence of the MM polymorphism at codon 129 [87]. FFI is characterized by medication-resistant insomnia, sleep fragmentation, disturbances of the autonomic nervous system, motor disorders, and progressive cognitive impairment [88]. The most affected areas are the mediodorsal and anterior ventral thalamic nuclei, followed by the pulvinar and the olives. Extensive neuronal loss and astrocytic gliosis are the main neuropathological findings, whereas spongiform transformations are missing [89]. FFI has not been found in the Czech Republic. 

### 1.4. Differential Diagnosis of Human Prion Diseases

The clinical picture in typical forms of TSEs is relatively specific, and with additional methods (MRI, cerebrospinal fluid examination, RT-QuIC), a high degree of diagnostic certainty can be achieved. Nonetheless, the differential diagnosis needs to consider several main groups (neurodegenerative—pure or comorbid, autoimmune including paraneoplastic, infectious, toxic/metabolic [88,90,91], and tumorous). Primary neurodegenerative diseases include Alzheimer’s disease (AD), frontotemporal dementia (FTD), dementia with Lewy bodies (DLB), corticobasal syndrome (CBS), multiple-system atrophy (MSA), motor neuron disease (MND), progressive supranuclear palsy (PSP), and normal-pressure hydrocephalus [89]. However, it is important to point out that “pure” forms of neurodegenerative diseases usually do not imitate CJD; however, comorbid cases can (see Table 5). The most common diagnoses possibly clinically mimicking CJD are AD, frontotemporal lobar degeneration (FTLD), DLB, and tauopathies, more often with, or without comorbid neurodegenerative disease, including less common comorbid neurodegenerations (FTLD+DLB, AD+FTD including Pick disease, AD+PSP). 

Paraneoplastic syndromes [92] in the form of paraneoplastic cerebellar degeneration or limbic encephalitis, autoimmune encephalitis [93], vasculitis [94], but also Hashimoto thyroiditis complicated by Hashimoto encephalopathy [95] must be included in the group of autoimmune processes that can mimic TSEs. Infectious forms are mainly caused by herpes simplex encephalitis.

Hepatic [96] or renal metabolic encephalopathies, as well as Wernicke-Korsakoff syndrome [97], which usually occurs in the chronically malnourished, especially ethylic patients, should be diagnostically considered in cases of rapidly progressive dementias. Clinically, these illnesses can have highly variable presentations.

We also noted cases of rapidly progressing dementia with atypical clinical symptoms and clinical suspicion of Creutzfeldt–Jakob disease in whom the subsequent neuropathological examination revealed the presence of a tumorous infiltration of the brain (primary, secondary, or lymphomatous) or meningeal carcinomatosis [98].

Although the neuropathological diagnosis of isolated prion disease is relatively straightforward, it is necessary to investigate possible co-pathologies in the brain that could modify the clinical, biochemical, and morphological manifestations observed antemortem. The influence of these modifying factors on the profile of biomarkers in cerebrospinal fluid is crucial [99]. 

### 1.5. CSF Biomarkers in TSE

RT-QuIC and 14-3-3 protein analysis are CSF biomarkers routinely used to confirm the diagnosis of probable CJD. 

#### 1.5.1. Protein 14-3-3 

The 14-3-3 proteins are highly expressed in the brain. They are located in the cytoplasmic compartment, intracellular organelles, and in the plasma membrane of neurons [100,101]. Detection of the 14-3-3 protein in the CSF is part of the WHO diagnostic criteria for probable sCJD [39,94]. There are seven isoforms of the 14-3-3 protein, only four of them have been detected in the CSF of sCJD patients, and only two (the β- and γ-isoform) seem to be suitable biomarkers for a differential diagnosis of sCJD [100]. This biomarker is not only present in the CSF of sCJD patients; it indicates rapid ongoing neuronal destruction in a variety of progressive neurological disorders [100,102]. Therefore, 14-3-3 positivity is not specific for sCJD, but the test should be indicated when the diagnosis of sCJD is considered. Total (t)-tau can also reflect neuronal destruction and is believed to correlate with the rate of axonal degeneration [103]. Since t-tau levels are dramatically increased in patients with prion diseases [103], we compared the ROC curves of 14-3-3β, t-tau, and their combinations in prion vs. non-prion neurodegenerative diseases. The presence of 14-3-3β was determined using a standardized Western blot protocol (used by all laboratories for the diagnosis of CJD) and followed EURO-CJD standards, with stringent quality control. We performed a standardized qualitative Western blot analysis for 14-3-3β in duplicates. Levels of t-tau were measured using commercially available enzyme-linked immunoassay (ELISA) kits (INNOTEST hTAU Ag, cat. #80323, Innogenetics/FUJIREBIO and Total-Tau ELISA, cat. #EQ 6531-9601-L, EUROIMMUN) according to the manufacturers’ instructions.

**Figure 1:**
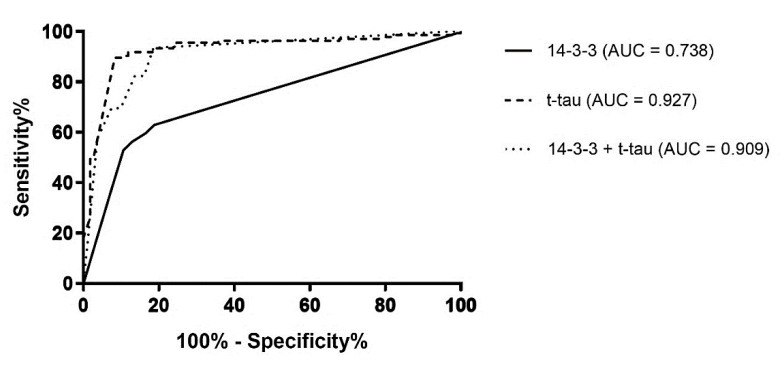
ROC diagrams for protein 14-3-3 positivity (solid line), t-tau values higher than 1200 pg/mL (dashed line), and the combination of protein 14-3-3 positivity and t-tau values higher than 1200 pg/mL (dotted line) in prion vs. non-prion disease cases. t-tau—total tau; AUC—area under the curve.

Comparing 14-3-3 positivity and t-tau levels in prion diseases vs. non-prion diseases using ROC curves, the AUC values were 0.738 and 0.927 (both *p* < 0.0001), respectively (Figure 1). For 14-3-3 positivity in prion diseases, the sensitivity was 63.1%, and the specificity was 81.1%. For t-tau, the cut-off was assessed to be 1200 pg/mL, with a sensitivity of 87.5% and a specificity of 91.5%. When both variables were taken together, the AUC was 0.909 (*p* < 0.0001), which gave the highest sensitivity (93.2%) but the lowest specificity (66.7%). Our results indicate that t-tau levels or t-tau levels combined with 14-3-3 positivity work better for detecting ongoing prion disease than 14-3-3 positivity alone, which we previously described [104]. Total tau can be a helpful tool in the differential diagnosis of sCJD and thus should be measured in addition to 14-3-3, especially when RT-QuIC is unavailable due to technical reasons and/or cost [105].

#### 1.5.2. RT-QuIC 

Real-time quaking-induced conversion (RT-QuIC) is used to demonstrate the ability of PrP^Sc^ (present in the CSF) to initiate the conversion of physiological PrP^C^ into a pathological conformation in which the β-sheet pattern predominates with a marked tendency toward real-time aggregation [106]. Imaging is performed by binding to thioflavin T, which emits fluorescence that can be immediately detected [106]. The method was introduced in 2010 and enabled detection of small amounts of PrP^Sc^ in approximately 90 h [106], with high levels of specificity (100% specificity, 95.8% diagnostic sensitivity), which is in contrast to other CSF biomarkers [107]. Second-generation RT-QuIC (called QuIC CSF or IQ-CSF), which uses truncated hamster PrP as a substrate, maintains a specificity of 98–100%; however, the reaction time is reduced to 30 h [108,109,110,111]. Moreover, RT-QuIC has the ability to detect PrP^Sc^ in olfactory mucosa samples or in a skin biopsy. RT-QuIC allows an accurate and rapid diagnosis, which is significant in the differential diagnosis of treatable diseases mimicking TSEs; however, this method has not yet been introduced into clinical practice and remains available only for scientific purposes in the Czech Republic.

### 1.6. Definite Diagnosis of Human Prion Diseases

A definitive diagnosis of TSE is based on the detection of PrP^Sc^ in brain tissue usually from brain autopsy [94]. Because Creutzfeldt–Jakob disease is not a high priority in differential diagnostic considerations, brain biopsy should be reserved for the search of treatable causes of progressive dementia. After confirmation of prion disease by Western blot and immunohistochemical methods, molecular genetic testing is routinely performed, aimed at detecting polymorphisms of codon 129 and possible mutations in the *PRNP* gene in genetic forms. About 15% of prion diseases have a hereditary basis, and in many cases, even in the absence of a relevant family history [77].

Cases of CJD are then divided according to the polymorphism at codon 129 to the three categories: MM homozygotes, VV homozygotes, and MV heterozygotes. According to the size of the proteinase K-resistant core of PrP^Sc^ (21 and 19 kDa), type 1 and type 2 of PrP^Sc^ is recognized, however, by combination of these techniques we only achieve a division into six subcategories: MM1, MM2, MV1, MV2, VV1, and VV2. Based on histopathological criteria, the subcategory MM2 is subsequently divided into MM2-cortical (MM2-C) and MM2-thalamic (MM2-T) form with characteristic major changes in thalamus and olives [32]. Similarly, subsequent neuropathological criteria for the MV2 cases exists. Predominantly cortical (MV2C) and predominant kuru plaques form (MV2K) are recognized; moreover, a histotype (MV2K+C) sharing both characteristics is also described [112]. 

Each histotype has its own characteristic histological picture:

MM1/MV1:

- Spongiform degeneration: formed by fine vacuoles predominantly affecting corticostriatal-thalamic and cerebellar areas, whereas hippocampal region is relatively spared.
- PrP deposits: prevailing synaptic location.

MM2/MV2C:

- Spongiform degeneration: large confluent vacuoles.
- PrP deposits: predominant involvement of cortex and subiculum, minor involvement of brainstem with cerebellum.

MV2K:

- PrP deposits: kuru-like plaques mainly in cerebellar granular layer, plaque-like deposits in other cortical regions.

VV1:

- Spongiform degeneration: medium-sized vacuoles involving cortex, striatum, beside spared cerebellar region.
- PrP deposits: synaptic pattern.

VV2:

- Spongiform degeneration: fine or medium-sized vacuoles; more severe involvement of subcortical grey matter by comparison with cerebral neocortex, and of hippocampus plus subiculum by comparison with occipital cortex.
- PrP deposits: cerebellar plaque-like structures and perioneuronal pattern in deep cortical layers and hippocampal region.
- Others: cerebellar atrophy.

MM2T (sFI):

- Spongiform degeneration: absent in cerebellum.
- Others: moderate to severe selective atrophy of thalamus and olives.

MVK+C:

- Spongiform degeneration: extensive vacuolarization.
- PrP deposits: same as MV2K (kuru-like plaques mainly in cerebellar granular layer, plaque-like deposits in other cortical regions), in addition with perivacuolar and coarse PrP deposits in the grey matter [113].

Upon detection of PrP^Sc^, the examined tissue is first digested with proteinase K, which degrades physiological PrP^C^. Subsequently, monoclonal antibodies directed against PrP recognize pathological PrP^Sc^ molecules that are resistant to proteinase K digestion. Indirect immunohistochemical methods or Western blot are used as a standard, although RT-QuIC should not be omitted from the list of diagnostic methods.

#### 1.6.1. Western Blot 

Western blot (WB) is more sensitive and faster than immunohistochemical methods. The fixation time of the whole brain is about 3–4 weeks, after which a neuropathological examination can be performed.

The time from autopsy to a definitive diagnosis, including the exclusion of other possible neurodegenerative diseases, is therefore around six weeks but can take longer. WB makes it possible to demonstrate the presence of PrP^Sc^ on the second day after autopsy. For WB analysis, the frontal lobe is routinely analyzed. Nonetheless, WB analysis provides the basic information about the presence of PrP^Sc^ and can only distinguish types (1, 2, 1+2 or 3) of PrP^Sc^. For complete information about the PrP^Sc^ strain, other methods must be performed (see below). 

##### Western Blot in Corneal Pre-Transplantation Testing

Since the retina and optic nerve are extensions of the central nervous system, the WHO considers them to be a category I: high-infectivity tissue. In the Czech Republic, approximately 500 corneal transplants are carried out each year. Since January 2007, testing all corneal donors for PrP^Sc^ is mandatory with the goal of increasing the safety of corneal transplants. For the detection of PrP^Sc^ in brain tissue by Western blot, the frontal lobe is routinely analyzed according to WHO guidelines. Complete neuropathological work-up is not performed in corneal donors‘ brain tissue. Except for a few cases of CJD [114,115], no other neuropathies were determined to be transferred during the corneal transplantation to our knowledge. All tests are performed exclusively by the immunological laboratory at the NRL, which cooperates with all eye banks in the Czech Republic. During this time (2007–2020), 6590 samples were tested; all were negative, four were suspected of having PrP^Sc^, but after further investigations, they were also found to be negative. Traceability of donors, through the National Donor Register and the National Transplant Register, for a period of 30 years is ensured by the Transplantation Act [116].

To the best of our knowledge, the Czech Republic is the only country in the world where legislation requires Western blot tests of brain tissue from every eye tissue donor. [117]. 

In neighboring Slovakia, where gCJD accounts for 74.2% of cases (compared to 16.28% in the Czech Republic) [118], determination of the codon 129 genotype together with detection of the E200K mutation is the method of choice for corneal donors testing [119]. 

#### 1.6.2. Immunohistochemistry

An immunohistochemical examination determines the distribution of PrP^Sc^ in different parts of the brain as well as the morphology of immunoreactive structures since PrP^Sc^ may occur in the form of plaques, plaque-like structures or diffuse synaptic deposits.

According to WHO criteria, one of the above methods is enough to confirm a definitive diagnosis of prion disease, although a combination of both is recommended.

A complete definitive diagnosis of prion disease has three parts, i.e., neuropathological, immunological (Western blot), and molecular genetics. Only a combination of these approaches can provide a definitive diagnosis of sporadic or genetic prion disease. 

#### 1.6.3. Genetic Testing 

Among the diagnosed cases in the Czech Republic, the distribution of polymorphisms at codon 129 is 60% methionine homozygosity (MM), 24% methionine/valine heterozygosity, and 16% valine homozygosity (VV); additionally, approximately 16% of cases are due to an inherited mutation in the *PRNP* gene. 

A common coding polymorphism at codon 129 in *PRNP* between methionine and valine (c.385A>G) plays a critical role in susceptibility to prion diseases with homozygotes (i.e., MM or VV) being at higher risk. Easier dimerization and oligomerization of PrP homozygotes compared to heterozygotes is an important factor in the pathogenesis of prion diseases.

In total, approximately 50 mutations of the *PRNP* gene have been described around the world. The most common mutations in the *PRNP* gene in the Czech population are E200K, D178N, R208H, and P102L, as well as del/ins in the repetitive sequence. The general penetration of these mutations increases with age, and in some populations reaches 100% after the age of 85; in others, for example, in Slovakia or Italy, penetration is up to 60% [118].

The E200K mutation is the most widespread in the Czech Republic (30 cases), as well as in Slovakia, from where it was transferred to the Czech Republic. 

The D178N mutation was found in two cases. The clinical phenotype in those cases was influenced by the polymorphism at codon 129 since the D178N mutation with valine at codon 129 causes gCJD. If methionine is present at codon 129, fatal familial insomnia develops. The genetic background of D178N could be clinically missed; for genetic counselling purposes, however, the mutation can be detected from samples of the archived tissue [120].

The P102L mutation was found in eight cases, and no clinically specific symptoms for this mutation were observed, although we described a case mimicking gCJD [121].

The R208H mutation is very rare, and one family harboring this mutation was discovered in the Czech Republic in relation to a case mimicking progressive supranuclear palsy [122].

Del/ins of two or more octapeptide repeats is considered pathogenic since octapeptide repeat insertions increase the rate of protease-resistant PrP formations. The clinical phenotype is highly variable, often blending features of both CJD and GSS disease or sometimes even lacking specific histopathological changes. Patients with insertional mutations usually display signs of illness early in life that last for several years. The molecular basis for this phenotypic heterogeneity remains elusive but seems to depend on the size of the base pair insertion and the codon 129 polymorphism.

## 2. Human Prion Diseases in the Czech Republic 2001–2020—Results from a Nation-Wide Survey

### 2.1. Numbers of Detected Cases 

Autopsy verification of all suspected prion diseases is mandatory in the Czech Republic and is governed by hygienic-epidemiological surveillance. Data obtained regarding possible cases from clinical neurologists and subsequently confirmed cases are registered with the National Reference Laboratory (NRL) for Human Prion Diseases at the Department of Pathology and Molecular Medicine, Thomayer University Hospital, Prague, CZ. Autopsies are provided by the NRL [123] with clinically suspected neurodegenerations making up about one-third of performed autopsies done in the department. The NRL is the only reference center for neuropathology verification of prion disease in the Czech Republic so that the data represents the official results of prion surveillance in our country and consists of definitively neuropathologically confirmed cases.

**Figure 2:**
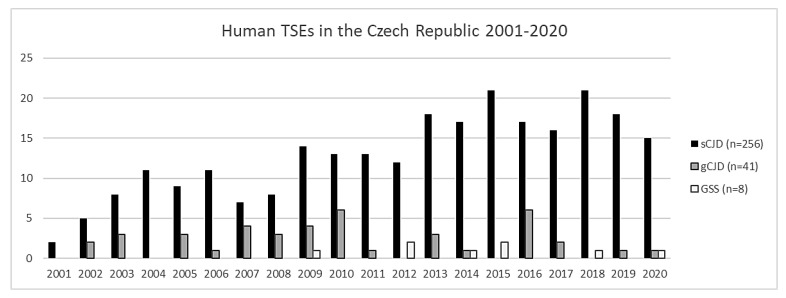
Number of patients who died with neuropathologically confirmed diagnose of prion disease in the Czech Republic (2001–2020). The legend shows the total number of cases for each type of prion disease.

From 2001 to 2020, a total of 305 cases of prion diseases were definitively confirmed, of which 256 cases were sCJD, 41 cases were gCJD, and 8 cases were GSS (see Figure 2), which corresponds to a total prevalence of approximately 15.25 cases per year. No cases of vCJD or FFI have ever been detected in the Czech Republic. Not all the brain samples referred to the NRL as possible/probable CJD were confirmed as definite CJD. Many other diagnostic entities were detected during autopsy, including neurodegenerations (in most cases comorbid), tumors, and autoimmune disorders (Table 5).

There is an apparent trend towards an increasing prevalence of TSEs due to the optimization of diagnostic methods, and in part by the fact that the NRL is headquartered at the Department of Neurology at Thomayer University Hospital, which provides consultations intra vitam in suspected cases for the whole of the Czech Republic. The increasing prevalence is well illustrated by the fact that in the first ten years (2001–2010) of the NRL’s existence, 115 cases of human prion diseases were detected, 88 of which have no hereditary background. However, over the next ten years (2011–2020), the number of confirmed TSE cases increased to 190, with 168 sporadic cases. This was an increase of 90.91% in detected sporadic cases over ten years, according to the predicted increase of diagnosed cases if surveillance was more intensive [124].

Nevertheless, during the COVID-19 pandemic in 2020, we noticed a slight decrease in the number of diagnosed patients suffering from TSEs; it was the fewest detected cases of sCJD since 2012. We speculate that this could be partly due to the greater burden on hospitals during the pandemic, which led to a reduction in TSE surveillance. 

**Figure 3:**
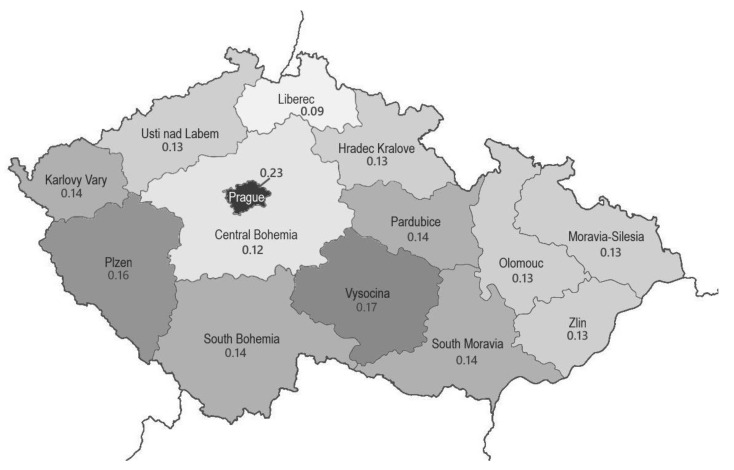
The incidence map of TSEs in individual regions of the Czech Republic. For each region, the number of cases per 100,000 population per year is indicated.

Considering incidence, in individual regions it ranges from 0.9 to 2.3 cases per million inhabitants (Figure 3), which is consistent with the worldwide numbers. The differences between individual regions are usually not significant and correlate with the existence of specialized neurological centers in the area. The age range in our cohort of patients is 39–69 years in GSS, 46–74 years in gCJD and 40–87 years in sCJD, which corresponds to a median age of 60.5 years in GSS, 56.5 years in gCJD and 67 years in sCJD. Thus, patients with the sporadic form have a higher median age than patients with genetically based TSEs (see Supplementary Material). 

### 2.2. CJD in Comorbidity 

There are many reports indicating that CJD very often occurs in comorbidity with other neurodegenerative diseases. Kovacs et al. [125] stated that the most common comorbidities with sCJD are tauopathies, such as primary age-related tauopathy, aging-related tau astrogliopathy, and argyrophilic grain disease (PART, ARTAG, and AGD, respectively). Our experience also supports these observations; in our cohort, approximately 62% of patients had sCJD or gCJD with a comorbid tauopathy, most often PART. The next most common comorbidity was Alzheimer’s disease, of which there are twice as many as pure CJD cases. Less common combinations include CJD+FTLD and CJD+DLB, which were rarely observed. We investigated the genetic background of the various comorbidities; however, our pilot study failed to find any important relationships [126].

### 2.3. Brain Biopsy 

During our 20 years of experience, a brain biopsy was rarely indicated. As mentioned above, brain biopsies are reserved for cases with a clinical suspicion of CJD associated with iatrogenic transmission (resulting from irreversible contamination of surgical instruments), and since CJD is incurable, a brain biopsy is the ultimum refugium in cases where the differential diagnosis includes a treatable disease. 

## 3. Conclusions 

At present, epidemiological surveillance of prion diseases in the Czech Republic is at a level comparable to other developed countries, and at the top with regard to systematic screening for PrP^Sc^ in the brain tissue of all corneal donors; the Transplantation Act, which mandates this screening, is unique in the world. The greater knowledge of clinicians and the routine use of magnetic resonance imaging and cerebrospinal fluid analysis has led to an increase in the detection of human prion diseases. In the Czech Republic, 16.28% of cases are hereditary; therefore, subsequent genetic consultation with the deceased patients’ relatives has become an important part of comprehensive approach to affected families. Although neither iatrogenic nor vCJD have been detected in the Czech Republic, the risk remains, mainly in connection with the increase in mini-invasive neurosurgical procedures and transmission via unscreened blood derivatives. The longstanding experience of our National reference center and the neuropathological feedback provided to various hospitals having referred patients with possible/probable CJD for autopsy progressively contribute to a better awareness and knowledge about the diagnostic clinical criteria and biomarkers of human prion diseases in our country.

## Figures and Tables

**Table 1:** Diagnostic criteria for definite, probable and possible sCJD.

**Possible** Creutzfeldt–Jakob disease: Rapidly progressive dementia with at least two of the following symptoms: myoclonus, cerebellar or visuospatial dysfunctions, pyramidal and/or extrapyramidal signs, akinetic mutism, and duration less than 2 years.
**Probable** Creutzfeldt–Jakob disease: Fulfilled criteria for possible CJD with: periodic sharp wave complexes at EEG, or 2. caudate/putamen hypersignal on magnetic resonance imaging (MRI) brain scan or at least two cortical regions (temporal, parietal, occipital) either on diffusion-weighted imaging (DWI) or fluid-attenuated inversion recovery (FLAIR) [41], or 3. positive cerebrospinal fluid 14-3-3 protein test, or 4. positive real-time quaking-induced conversion (RT-QuIC) in cerebrospinal fluid or other tissues.
**Definite** Creutzfeldt–Jakob disease: Progressive neurological syndrome and either neuropathological, immunocytochemical or biochemical confirmation.

**Table 2:** Diagnostic criteria for probable and definite iCJD.

**Probable** iatrogenic Creutzfeldt–Jakob disease diagnosis: progressive cerebellar syndrome in a recipient of human cadaver-derived pituitary hormone; or probable CJD with a recognized iatrogenic risk.
**Definite** iatrogenic Creutzfeldt–Jakob disease diagnosis: Definite CJD with a recognized iatrogenic risk.

**Table 3:** Diagnostic criteria for suspected and definite vCJD.

**Suspected** Variant CJD: current age or age at death less than 55 years psychiatric symptoms at illness onset and/or persistent painful sensory symptoms (frank pain and/or dysesthesia) dementia, and development ≥4 months after illness onset of at least two of the following five neurologic symptoms: impairment in coordination, myoclonus, chorea, hyperreflexia, or visual signs (if persistent painful sensory symptoms exist, ≥4 months delay in the development of the neurologic signs is not required) a normal or an abnormal EEG, but not the diagnostic EEG changes seen in sporadic CJD duration of illness of more than 6 months routine investigations of the patient do not suggest an alternative, non-CJD diagnosis no history of receipt of cadaveric human pituitary growth hormone or a dura mater graft no history of CJD in a first degree relative or prion protein gene mutation in the patient
**Definite** Variant CJD: Neuropathologic examination of brain tissue is required to confirm a diagnosis of Variant CJD. The following confirmatory features should be present. numerous widespread kuru-type amyloid plaques surrounded by vacuoles in both the cerebellum and cerebrum (florid plaques) spongiform change and extensive prion protein deposition shown by immunohistochemistry throughout the cerebellum and cerebrum

**Table 4:** Diagnostic criteria for probable and definite gCJD.

**Probable** genetic Creutzfeldt–Jakob disease diagnosis: probable CJD and confirmed/probable CJD in a first-degree relative, a neuropsychiatric disorder plus a disease-specific prion protein gene (*PRNP*) mutation.
**Definite** genetic Creutzfeldt–Jakob disease diagnosis: definite CJD with a recognized pathogenic *PRNP* mutation, and definite or probable TSE in a first-degree relative.

**Table 5:** Autopsy findings of non-prion diseases from brain tissue samples described as possible/probable CJD (2001–2020).

**Neurodegenerative disorders** **(usually comorbid)**	Alzheimer’s disease	88
	Frontotemporal dementia	66
	Dementia with Lewy bodies	27
	Progressive supranuclear palsy	8
	Multiple system atrophy	4
	Corticobasal degeneration	2
	Parkinson disease	1
**Neuroinfection and autoimmune diseases**	Encephalitis	20
	Malignant multiple sclerosis (Marburg variant)	2
**Ischemic and anoxic conditions**	Subcortical vascular dementia	7
	Post-anoxic encephalopathy	7
**Tumors**	Primary CNS lymphoma	5
	Gliomatosis cerebri	1
	Meningeal carcinomatosis	1
	Metastatic carcinoma	1
**Metabolic encephalopathy**	Wernicke-Korsakoff	5
**Others**	Subdural hematoma	1

## Data Availability

The authors confirm that all data underlying the findings are fully available without restriction. All data are included within the manuscript.

## Supplementary Materials

The following are available online at https://www.mdpi.com/article/10.3390/diagnostics11101821/s1.
